# Vitamin D Induces Differential Effects on Inflammatory Responses During Bacterial and/or Viral Stimulation of Human Peripheral Blood Mononuclear Cells

**DOI:** 10.3389/fimmu.2020.00602

**Published:** 2020-04-07

**Authors:** Jeremy Anderson, Lien Anh Ha Do, Zheng Quan Toh, Edwin Hoe, Andrea Reitsma, Kim Mulholland, Paul V. Licciardi

**Affiliations:** ^1^Murdoch Children's Research Institute, Melbourne, VIC, Australia; ^2^Department of Paediatrics, University of Melbourne, Parkville, VIC, Australia; ^3^Epidemiology and Public Health, London School of Hygiene and Tropical Medicine, London, United Kingdom

**Keywords:** pneumococcal, respiratory syncytial virus, vitamin D, inflammation, peripheral blood mononuclear cells

## Abstract

*Streptococcus pneumoniae* (pneumococcus) and respiratory syncytial virus (RSV) are the leading causes of respiratory infections amongst children <5 years of age. Co-infection with these pathogens is common during early life and often associated with increased disease severity. Epidemiological studies have shown that low levels of Vitamin D_3_ (VitD_3_) are associated with increased susceptibility to respiratory pathogens. However, the role of VitD_3_ in the context of pneumococcal and RSV exposure are poorly understood. We found that VitD_3_ significantly reduced Th17 cell expression and IL-17A and IL-22 secretion in peripheral blood mononuclear cells (PBMCs) when stimulated with a pneumococcal whole cell antigen (WCA). Levels of IFN-γ were also decreased whilst IL-10 and IL-1β were increased. Effects of VitD_3_ on innate responses following RSV stimulation was limited, only reducing IL-6. VitD_3_ also reduced the number of TLR2+CD14+ monocytes, whilst increasing TLR7+CD14+ monocytes and TLR4+CD56+ NK cells. In WCA-stimulated PBMCs, VitD_3_ increased IL-1β levels but reduced TLR2+CD14+ monocytes. For pneumococcal WCA-RSV co-stimulation, VitD_3_ only had a limited effect, mainly through increased IL-1β and RANTES as well as TLR4+CD56+ NK cells. Our results suggest that VitD_3_ can modulate the inflammatory response to pneumococci but has limited effects during viral or bacterial-viral exposure. This is the first study to examine the effects of VitD_3_ in the context of pneumococcal-RSV co-stimulation, with important implications on the potential role of VitD_3_ in the control of excessive inflammatory responses during pneumococcal and RSV infections.

## Introduction

*Streptococcus pneumoniae* (pneumococcus) and respiratory syncytial virus (RSV) are the leading causes of lower respiratory tract infections (LRTIs) amongst children and older adults (1, 2). Importantly, co-infections with these pathogens are becoming increasingly recognized as a major contributor to severe LRTIs requiring hospitalizations (3, 4). Innate responses to RSV or pneumococcal bacteria can prime the host for secondary infection by activating inflammatory cells such as macrophages and neutrophils (5, 6). T-helper cell 17 (Th17) responses are important in the control of pneumococcal colonization, which is a pre-requisite step in the development of invasive disease (7). However, chronic exposure can lead to dysregulated inflammatory responses and pathology. Balancing the inflammatory response during co-infection may be a strategy to reduce severe morbidity (8).

The discovery of the Vitamin D receptor (VDR) and cytochrome P450 27B1 (CYP27B1) enzyme expression on immune cells has driven exploration into whether the active metabolite of vitamin D, 1,25 dihydroxyvitamin D_3_ [1,25(OH)_2_D_3_] (VitD_3_), has immunoprotective properties in the context of infection (9–12). Numerous observational data and clinical trials suggest that insufficient VitD_3_ levels are associated with increased susceptibility to respiratory pathogens, and that VitD_3_ supplementation in high disease burden settings may be beneficial (13–15). Interactions between the VDR and VitD_3_ mediates anti-inflammatory effects on both the innate and adaptive immune systems, thereby regulating immunity in the context of bacterial and/or viral inflammation. Innate cell subsets including neutrophils, macrophages, and dendritic cells (DCs) all express VDR and respond to VitD_3_. Following contact with pathogens through binding to their toll-like receptors (TLRs), genes that encode up-regulation of the VDR and production of CYP27B1 become expressed (16). Stimulation of the VDR in these cells enhances their bactericidal, anti-microbial, chemotactic, and phagocytic capabilities (17). Similarly, VitD_3_ also influences the adaptive immune responses either directly or indirectly through DCs, altering their cytokine production. This influences Th17 activation and function, through increasing IL-10 and decreasing IL-17A secretion, which are associated with RSV and pneumococcal infections (18).

In this study, we examined the effect of VitD_3_ on inflammatory responses in the context of pneumococcal and RSV co-stimulation. We treated peripheral blood mononuclear cells (PBMCs) isolated from healthy adults with VitD_3_ and stimulated with either pneumococcal whole cell antigen (WCA), RSV or WCA-RSV together to study host cytokine responses and the frequency of key immune cell populations important during pneumococcal and RSV infections, focusing on Th17 (for pneumococcus) and innate (for RSV alone and pneumococcal-RSV co-infection) inflammatory responses. Our results provide evidence that VitD_3_ reduces pneumococcal Th17 responses, but had limited effects in modulating the inflammatory response during pneumococcal-RSV co-stimulation. These findings are important in the context of novel strategies such as VitD_3_ supplementation to reduce the severity and incidence of both pneumococcal and RSV infections in VitD_3_ in high risk populations.

## Methods

### Study Samples

Twelve healthy adults aged from 19 to 64 years old were enrolled into the study. A single blood sample (~20 mLs) was collected from each individual into a sodium heparin tube. All subjects gave their informed consent and the study was approved by the Royal Children's Hospital Human Research Ethics Committee (HREC).

### Materials

The active metabolite of VitD_3_ was purchased from Tocris Bioscience (Bristol, UK). The pneumococcal whole cell antigen (WCA) was kindly provided by PATH under a Materials Transfer Agreement. Live RSV-A2 strain and A549 cell line was purchased from American Type Culture Collection (ATCC; Virginia, USA).

### RSV Preparation

For RSV stock preparation, RSV A2 strain was grown in A549 cells and purified by centrifugation through 30% sucrose layer as described previously (19). The harvested virus was collected in DMEM culture medium containing 20% sucrose and aliquoted, then snap-frozen and stored at−80°C until subsequent experiments. The titre of purified virus stocks were determined by plaque assay according to a previous method (20).

### PBMC Culture

The PBMCs were isolated immediately after blood collection by density gradient centrifugation using Lymphoprep (Axis-Shield, Oslo, Norway) at 400 × g for 30 min without brake at room temperature (RT). Isolated PBMCs were then washed twice in RPMI-1640 medium (Sigma-Aldrich, St. Louis, USA) supplemented with 10% FBS, 1,000 IU penicillin-streptomycin and 200 nM L-glutamine (RPMI-FBS) at 500 × g for 10 min at RT. The cells were resuspended in 1 mL RPMI-FBS and the cell concentration was determined using the Trypan blue exclusion dye (Sigma-Aldrich, St. Louis, USA) method where a 1:1 mixture of PBMCs and Trypan Blue dye was added to a haemocytometer (Neubauer chamber) and counted under a microscope. For pneumococcal assays, 1 × 10^6^ PBMCs/mL were pre-treated with 100 nmol/L VitD_3_ for 24 h then stimulated with 1 μg/mL WCA for 5 days. Based on a model adapted from previous studies (21), 1 × 10^6^ PBMCs/mL in our co-stimulation experiments were incubated simultaneously with live RSV (MOI = 1) and 1 μg/mL WCA and 100 nmol/L VitD_3_ for 24 h. Supernatants were then harvested and stored at−30°C until use.

### Cytokine Measurement

Levels of IFN-γ, IL-8, IL-10, TNF-α, MCP-1, and RANTES in cultured supernatants from PBMCs were measured using a human cytokine multiplex bead array method as per the manufacturer's instructions (Milliplex; Millipore Corporation, Billerica, MA, USA). IL-6, IL-17A, and IL-22 were measured using commercial ELISA kits according to the manufacturer's instructions (R&D Systems; Minneapolis, Minnesota, USA).

### Flow Cytometry

To identify specific immune cell subsets in PBMCs following VitD_3_ treatment, cells were stained with fluorescently-conjugated monoclonal antibodies; CD4-BUV737, CD45RO-APC, CD161-FITC, CD194-V450, CD196-PE, CD14-BV605, CD19-APC-H7, CD56-BV421, CD282-AF647 (TLR2), CD284-BV786 (TLR4; all from BD Bioscience; San Diego, CA, USA), and anti-TLR7-PE (Gibco Life Technologies, Carlsbad, USA), Zombie Aqua™ Fixable Viability Kit (BioLegend, San Diego, USA). Compensation bead particles were used to account for spectral overlap (BD Bioscience, San Diego, CA, USA) and analyzed using the BD LSRII flow cytometer. Unstained PBMCs and fluorescence minus one (FMO) were used as controls and a minimum of 100,000 events were analyzed per sample gated on live, single cell lymphocyte gate based on FSC and SSC, where the expression of the cell surface molecules was evaluated using FlowJo, LLC v10.4.2 software. Refer to Supplementary Figures 1–4 for gating strategies.

### Statistical Analysis

Data is presented as median ± IQR for cytokine and flow cytometry results. Comparison of VitD_3_ treated and untreated cytokine responses and cell populations were determined using a paired non-parametric, Wilcoxon sign-rank test. The data was graphically represented and statistically analyzed using Graphpad prism 6 software (Graphpad Software Inc, California, USA). All tests performed were two-tailed and a *p* < 0.05 was considered significant.

## Results

### VitD_3_ Reduces Inflammatory Cytokines and Th17 Frequency in PBMCs Following Pneumococcal Stimulation

**Figure 1:**
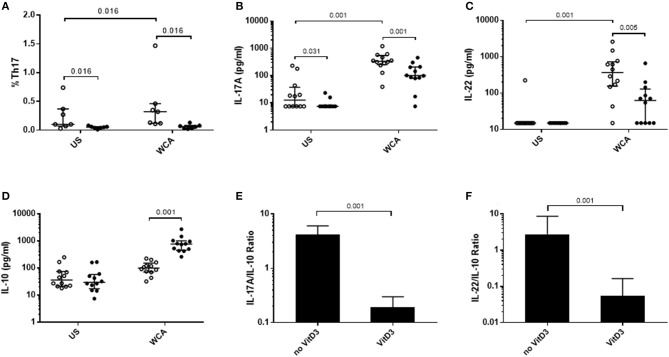
*VitD*_3_ *decreases pneumococcal Th17 responses*. 1 × 10^6^ PBMCs/mL were pre-treated with 100 nmol/L VitD_3_ for 24 h, prior to stimulation with 1 μg/mL WCA for 5 days. Th17 frequency **(A)** was measured by flow cytometry. Th17 populations were determined by positive staining for cell surface markers obtained from live single lymphocytes. These were considered CD4+CD45+CD161+CD194+CD196+. IL-17A **(B)**, IL-22 **(C)**, IL-10 **(D)**, IL-17A-IL-10 ratio **(E)**, IL-22-IL-10 ratio and **(F)**, concentrations were measured by an ELISA in pg/mL. Open circles represent untreated PBMCs, whilst closed circles represent VitD_3_ treated PBMCs. Data shown represents median ± IQR; *n* = 12 per group for cytokine analyses and *n* = 8 per group for flow cytometry.

We examined the potential for VitD_3_ to modify Th17 responses as this is important in the control of pneumococcal colonization. Pneumococcal WCA was used as this has previously been shown to specifically induce Th17 responses in mice and humans (22, 23). Stimulation of PBMCs with pneumococcal WCA significantly enhanced the proportion of Th17 cells and levels of Th17-related cytokines IL-17A and IL-22 (Figures 1A–C). VitD_3_ significantly reduced the Th17 frequency in both unstimulated and WCA-stimulated PBMCs (both *p* = 0.016; Figure 1A), as well as IL-17A and IL-22 in WCA-stimulated cells compared with untreated cells stimulated with WCA (both *p* < 0.01; Figures 1B,C). In contrast, VitD_3_ increased IL-10 in WCA-stimulated PBMCs (*p* = 0.001; Figure 1D). Consistent with these findings, the IL-17A/IL-10 and IL-22/IL-10 ratios (both *p* = 0.001) were also significantly decreased by VitD_3_ (Figures 1E,F).

**Figure 2:**
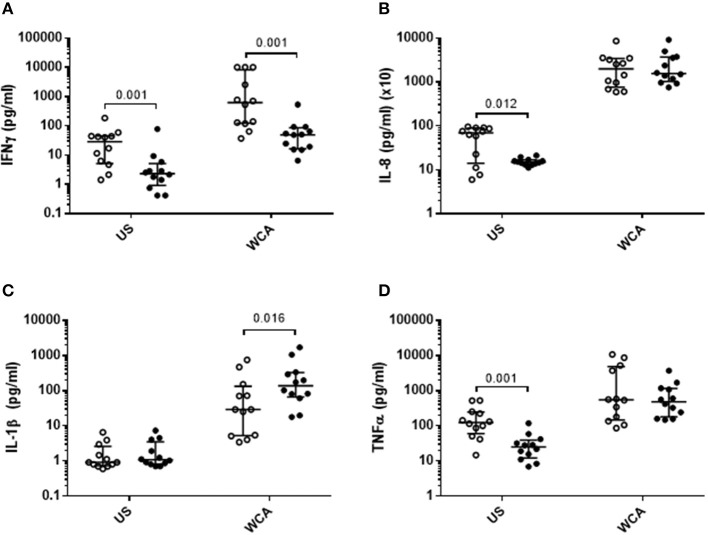
VitD_3_ reduces pneumococcal pro-inflammatory cytokines and increases anti-inflammatory cytokines. 1 × 10^6^ PBMCs/mL were pre-treated with VitD_3_ (100nmol/L) for 24 h, prior to stimulation with 1 μg/mL WCA for 5 days. **(A)** IFN-γ, **(B)** IL-8, **(C)** IL-1β, and **(D)**. TNF-α concentrations were measured by a multiplex assay in pg/mL. Open circles represent untreated PBMCs, whilst closed circles represent VitD_3_ treated PBMCs. Data shown represents median ± IQR; *n* = 12 per group.

VitD_3_ also significantly reduced the level of IFN-γ, IL-8, and TNF-α in unstimulated cells (Figures 2A,B,D), while in WCA-stimulated cells, VitD_3_ significantly reduced IFN-γ while increasing IL-1β (Figures 2A,C) but did not alter IL-8 or TNF-α levels (Figures 2B,D).

### VitD_3_ Does Not Modulate Inflammatory Responses During Pneumococcal-RSV Co-stimulation

**Figure 3:**
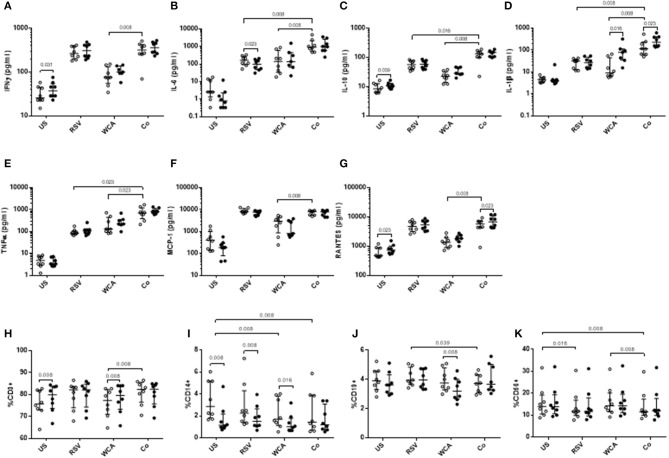
The effect of VitD_3_ on pro-inflammatory cytokines, chemokines, and immune cell subsets during bacterial-viral co-stimulation. 1 × 10^6^ PBMCs/mL were treated with VitD_3_ (100 nmol/L) at the time of stimulation. PBMCs were stimulated with live RSV at an MOI = 1, WCA (1 μg/mL) or WCA-RSV (designated “co”). **(A)** IFN-γ, **(B)** IL-6, **(C)** IL-10, **(D)** IL-1β, **(E)** TNF-α, **(F)** MCP-1, and **(G)** RANTES. For immune cell subsets cell populations were determined by positive cell surface staining from live single lymphocytes. **(H)** CD3+, **(I)** CD14+, **(J)** CD19+, **(K)** CD56+. Concentrations were measured by a multiplex assay in pg/mL. Open circles represent untreated PBMCs, whilst closed circles represent VitD_3_ treated PBMCs. Data shown represents median ± IQR; *n* = 8 per group.

To determine the effect of VitD_3_ in the context of pneumococcal and RSV co-stimulation, we undertook studies of the innate response using a model adapted from previous studies (21). Co-stimulation of PBMCs with WCA and RSV (WCA-RSV) resulted in increased cytokine responses for IL-6, IL-10, IL-1β, and TNF-α compared to RSV alone (Figures 3B–E). Compared to WCA stimulation, WCA-RSV significantly increased the level of all cytokines measured (Figures 3A–G). VitD_3_ treatment of WCA-RSV stimulated cells did not affect most cytokines, but increased the levels of IL-1β and RANTES (both *p* < 0.05; Figures 3D,G).

For RSV stimulation alone, VitD_3_ significantly reduced IL-6 only (*p* = 0.023; Figure 3B), but had no effect on any of the other cytokines measured. For WCA alone, VitD3 increased IL-1β only (*p* = 0.016; Figure 3E), consistent with our earlier observation (see Figure 1).

### Effect of VitD_3_ on Innate and Adaptive Cell Subsets and TLR Expression Following Pneumococcal-RSV Co-stimulation

To understand how VitD_3_ mediates these anti-inflammatory effects, we examined the phenotypic expression of key immune cell markers. We found that VitD_3_ treatment in the absence of any stimulation increased the frequency of CD3+ T cells (*p* = 0.008) while decreasing CD14+ monocyte populations (*p* = 0.039; Figures 3H–I) but had no effect on CD19+ or CD56+ cells (Figures 3J–K). When stimulated with WCA alone, VitD_3_ treatment significantly increased CD3+ cells (Figure 3H) but reduced CD19+ and CD14+ cell numbers compared with untreated PBMCs stimulated with WCA alone (Figures 3I,J), while for RSV alone, VitD_3_ only decreased CD14+ cells (Figure 3I). The CD14+ cell frequency was significantly decreased following WCA or WCA-RSV compared with unstimulated cells (*p* = 0.008 for both; Figure 3I).

VitD_3_ did not modulate the frequency of any of the immune cell populations in the context of WCA-RSV co-stimulation (Figures 3H–K). In the absence of VitD_3_, WCA-RSV co-stimulation significantly increased the percentage of CD3+ cells compared to WCA alone but reduced CD19+ cells compared to RSV alone (Figures 3H,J). Furthermore, CD56+ cells were reduced following stimulation with RSV alone compared with unstimulated cells while WCA-RSV reduced CD56+ cells compared to unstimulated and WCA alone-stimulated cells (*p* = 0.008 for both; Figure 3K).

**Figure 4:**
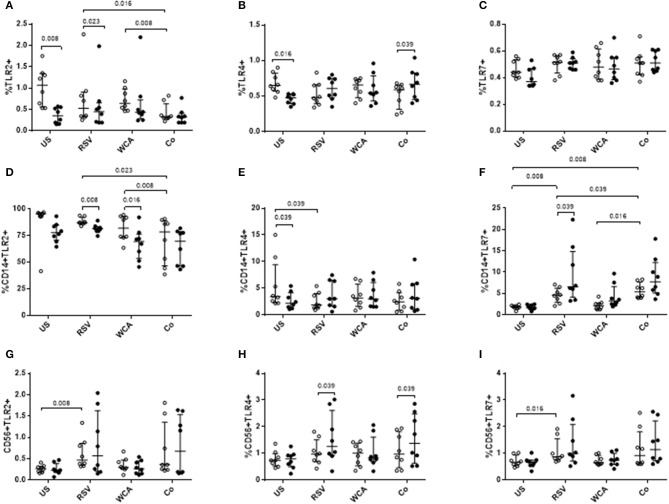
The effect of VitD_3_ on TLR expression in PBMCs. 1 × 10^6^ PBMCs/mL were treated with VitD_3_ (100 nmol/L) at the time of stimulation. PBMCs were stimulated with live RSV at an MOI = 1, WCA (1 μg/mL) or WCA-RSV (designated “co”) prior to phenotyping by flow cytometry. Cell populations were determined by positive staining for cell surface obtained from live single lymphocytes. **(A)** TLR2+, **(B)** TLR4+, **(C)** TLR7+, **(D)** CD14+TLR2+, **(E)** CD14+TLR4+, **(F)** CD14+TLR7+, **(G)** CD56+TLR2+, **(H)** CD56+TLR4+, **(I)** CD56+TLR7+. Open circles represent untreated PBMCs, whilst closed circles represent VitD_3_ treated PBMCs. Data shown represents median ± IQR; *n* = 8 per group.

We next examined TLR expression on PBMCs and specific cell populations to determine whether the effects of VitD_3_ is pathogen or ligand-specific (Figure 4). In unstimulated PBMCs, VitD_3_ significantly reduced the frequency of TLR2+ and TLR4+ cells but only TLR2+ cells following RSV stimulation (Figures 4A,B). TLR7 expression was not modulated by VitD_3_ for any of the conditions tested (Figure 4C). For WCA alone, VitD_3_ did not affect any TLRs. Co-stimulation with WCA-RSV significantly reduced the frequency of TLR2+ cells compared with WCA (*p* = 0.008) or RSV (*p* = 0.016) alone (Figure 4A). However, VitD_3_ only increased the percentage of TLR4+ cells when co-stimulated with WCA-RSV (*p* = 0.039; Figure 4B).

Co-stimulation with WCA-RSV significantly reduced the percentage of CD14+TLR2+ compared to RSV or WCA alone (Figure 4D). WCA-RSV had no effect on CD14+TLR4+ cells but significantly increased the frequency of CD14+TLR7+ cells compared with RSV or WCA alone or unstimulated cells (Figures 4E–F). However, VitD_3_ treatment significantly reduced CD14+TLR2+ frequency for RSV (*p* = 0.008) or WCA (*p* = 0.016) stimulation compared with either one alone but not WCA-RSV co-stimulation (Figure 4D). For CD14+TLR4+ cells, VitD_3_ only reduced the frequency in unstimulated cells (Figure 4E), while VitD_3_ significantly increased the percentage of CD14+TLR7+ cells for RSV alone (*p* = 0.039), with non-significant increases for both WCA alone and WCA-RSV conditions (Figure 4F). For CD56+ cells, RSV stimulation increased the percentage of TLR2 (*p* = 0.008) and TLR7 (*p* = 0.016; Figures 4G,I), VitD_3_ however only increased TLR4 frequency for RSV alone stimulation and WCA-RSV co-stimulation (*p* = 0.039) but did not alter TLR2 or TLR7 for any other condition tested (Figures 4G–I).

## Discussion

Co-infection with *S. pneumoniae* and RSV increases host inflammatory responses that often leads to severe respiratory disease requiring hospitalization. The anti-inflammatory effects of VitD_3_ are well-documented however their ability to modulate responses associated with bacterial or viral exposure are not well understood. This study extends our earlier observations of VitD_3_ effects on pneumococcal innate responses (24) to examine Th17 immunity and inflammatory responses following co-stimulation with pneumococcus and RSV. We found that VitD_3_ reduced pneumococcal Th17 inflammation but only had a limited effect on responses to WCA-RSV co-stimulation.

Pneumococcal exposure induces Th17 responses that protect against subsequent pneumococcal acquisition by enhancing recruitment of neutrophils and increasing anti-microbial peptide release through IL-17A and IL-22 secretion (25, 26). However, in low-and middle-income countries where pneumococcal carriage is high, a persistent IL-17A response occurs. The high level of exposure to pneumococcus in these settings limits the ability of IL-17A to reduce colonization, persisting to chronic inflammation through continued recruitment of neutrophils and macrophages (27). We used pneumococcal WCA as this is a well-characterized inducer of Th17 responses, and was a good model to examine the effects of VitD_3_ (7). We defined Th17 responses on the basis of CCR4 and CCR6 chemokine receptor expression as this has previously been described as a functional Th17 subset (28, 29). This was confirmed by the increased activation of Th17 cells and their related cytokines by WCA. VitD_3_ reduced the Th17 cell frequency and level of IL-17A and IL-22, suggesting a potent anti-Th17 effect. VitD3 also enhanced IL-10, lending support for the Th17-Treg axis (30, 31). This is important since unregulated Th17 responses can lead to significant inflammation and pathology. In view of our findings, VitD_3_ may have a critical role in maintaining the balance between these responses, particularly in populations where there is substantial exposure to pneumococcus during early life.

Increased levels of TNF-α and IL-6 during pneumococcal infection are associated with severity of disease (32). Pre-treatment with VitD_3_ decreased these cytokines while IL-1β were increased. This is consistent with a recent study by Sommer and Fabri (33), suggesting that VitD_3_ increases IL-1β to prime the innate response by influencing IL-1β gene transcription as no IL-1β is released prior to infection. This priming of the innate response by VitD_3_ is important to enhance anti-microbial activity through increased defensin-4 secretion (34–36). Further, studies to examine the production of antimicrobial peptides such as cathelicidins in response to VitD_3_ would be worthwhile.

To determine the role of VitD_3_ on the response to WCA-RSV co-stimulation, we used a different model to the Th17 studies to determine innate effects, based on a previous study (21). We observed increased cytokine responses in PBMCs following co-stimulation with WCA and RSV, recapitulating the effect seen during co-infection. Similarly, children given the live attenuated influenza vaccine had increased pneumococcal carriage, possibly due to increased CCR2 inflammatory monocytes which upregulate bacterial adherence receptors (37). Additionally, pneumococcal carriage also enhances secondary RSV infection. It has been shown that mice carrying pneumococcus prior to RSV infection exhibit much higher viral loads (38). Thus, the increase in IL-1β and RANTES levels by VitD_3_ is important as this enhances clearance of viral infections such as RSV by recruiting T cells and monocytes to the site of infection (39).

Monocytes and NK cells are crucial in the clearance of RSV, both contributing to type-1 interferon release to reduce pathology mediated by RSV (40, 41). We found that, VitD_3_ significantly reduced the number of CD14+ monocyte cells in response to WCA or RSV but not for WCA-RSV although a trend toward lower numbers was observed. In contrast, we found VitD_3_ had increased overall TLR4 expression as well as on CD56+TLR4+ cells following co-stimulation. TLR4 on innate cells such as monocytes and NK cells bind LPS which initiates pro-inflammatory responses to pneumococcus and may also interact with the F-protein on RSV, respectively. As macrophage depletion in secondary pneumococcal infection increases pneumococcal dissemination, increased TLR4 expression may be beneficial in reducing its capacity to spread (42–44). In CD14+ monocytes, both WCA and RSV stimulation alone had decreased TLR2 expression by VitD_3_, while total TLR2 and TLR4 populations were reduced by VitD_3_ in unstimulated cells. The expression of TLR2 on epithelial cells is upregulated following RSV infection and plays an important role in innate activation. TLR2 deficient mice show impaired neutrophil migration and pro-inflammatory cytokine production by macrophages, alongside uncontrolled RSV replication (45). Therefore, VitD_3_ appears to restore TLR2 responsiveness by upregulating VDR transcription factors to influence macrophage and neutrophil activity (42). VitD_3_ also increased CD3 expression on T cells while lowering CD19 expression on B cells for WCA only suggesting that VitD_3_ may also have important regulatory roles in terms of T cell differentiation and antibody production. Prior studies have demonstrated that VitD_3_ can modulate certain T-helper cell populations in the context of pneumococcal stimulation (18), but the implications on B cell function require further investigation. As we did not measure cell proliferation, we cannot rule out the possibility that the anti-inflammatory effect of VitD_3_ may also involve effects on proliferation.

Interestingly, we found that TLR7 on CD14+ monocytes and TLR4 on CD56+ cells was significantly upregulated by VitD_3_ for RSV and WCA-RSV, while TLR2 was reduced, suggesting that in response to pathogen encounter, VitD_3_ may differentially effect bacterial and viral pattern recognition receptors. RSV has a number of surface proteins that can bind directly with TLR4 and/or TLR7, as well as intracellular receptors such as RIG-I (46), suggesting that VitD_3_ may be important in regulating viral or viral-bacterial co-infection. Indeed, VitD_3_ has been shown to activate antiviral RIG-I pathways during rotavirus infection of pigs (47). While TLR7 is mainly expressed intracellularly, it has also been shown to be expressed on the surface of immune cells (48). Moreover, previous studies have shown that TLR7 responses are impaired in otherwise healthy individuals with low vitamin D levels (49). Recent evidence has shown that airway neutrophil influx following RSV infection mediates anti-bacterial effects in relation to pneumococcus (50). We previously found that VitD_3_ was able to reduce neutrophil migration (24), but how VitD_3_ might regulate this response at the respiratory mucosa during RSV-pneumococcal infection is an important question that remains unanswered.

Respiratory infections are most prevalent during winter when VitD_3_ status is lowest in individuals. Multiple epidemiological studies have suggested VitD_3_ deficiency to be an associated risk factor for susceptibility to respiratory diseases (51, 52). Novel strategies to prevent and/or reduce pneumococcal inflammatory responses are important in the context of secondary viral infections and disease (53). The effect of VitD_3_ in co-infection models is unknown, and further research into the potential benefits are required (54, 55).

Our study has several limitations, the main one being the small sample size. Despite this, we were still able to demonstrate the anti-inflammatory effects of VitD_3_, similar to other studies (21, 56). While we did not measure VitD_3_ status in our cohort, we have previously shown that most adults in our setting are VitD_3_ insufficient (24) which may resemble to some extent the VitD_3_ status in other geographical settings. Our results also need to be interpreted with caution as we examined VitD_3_ effects in adults which may not directly translate into a pediatric population (57). Further, studies in high burden settings or high risk groups [e.g., preterm infants; (58)] are required to fully investigate the role of VitD_3_ to protect against severe respiratory infection during early life.

## Conclusion

Our results suggest that VitD_3_ has important biological effects in the context of bacterial stimulation but was less effective for bacterial-viral co-stimulation, through modulation of innate and adaptive responses important for protection. This effect of VitD_3_ was associated with its effects on Th17 cells as well as expression of TLR responses on key innate cells. Populations most at risk from respiratory infection are generally VitD_3_ deficient, and this is associated with increased cytokine responses that promote disease severity. Future studies should aim to examine the effect of VitD_3_ during co-infection, using PBMCs from pediatric cohorts to better determine the potential efficacy of VitD_3_ trials in high-risk populations.

## Data Availability Statement

The datasets generated for this study are available on request to the corresponding author.

## Ethics Statement

The studies involving human participants were reviewed and approved by Human Research Ethics Committee, Royal Children's Hospital, Melbourne, Australia. The patients/participants provided their written informed consent to participate in this study.

## Author Contributions

JA, LD, and PL conceived the study design and prepared the first draft of the manuscript. JA, ZT, EH, and AR performed the experiments. KM provided advice on study and edited the manuscript. All authors edited and approved the final version of the manuscript.

### Conflict of Interest

The authors declare that the research was conducted in the absence of any commercial or financial relationships that could be construed as a potential conflict of interest. The reviewer RL declared a shared affiliation, though no other collaboration, PL, LD, and ZT to the handling editor.

## References

[1] O'Brien KL Wolfson LJ Watt JP Henkle E Deloria-Knoll M McCall N. Burden of disease caused by *Streptococcus pneumoniae* in children younger than 5 years: global estimates. Lancet. (2009) 374:893–902. 10.1016/S0140-6736(09)61204-6 19748398

[2] Shi T McAllister DA O'Brien KL Simoes EAF Madhi SA Gessner BD. Global, regional, and national disease burden estimates of acute lower respiratory infections due to respiratory syncytial virus in young children in 2015: a systematic review and modelling study. Lancet. (2017) 390:946–58. 10.1016/S0140-6736(17)30938-8 28689664 PMC5592248

[3] Cebey-Lopez M Pardo-Seco J Gomez-Carballa A Martinon-Torres N Martinon-Sanchez JM Justicia-Grande A. Bacteremia in children hospitalized with respiratory syncytial virus infection. PLoS ONE. (2016) 11:e0146599. 10.1371/journal.pone.0146599 26872131 PMC4752219

[4] Weinberger DM Klugman KP Steiner CA Simonsen L Viboud C. Association between respiratory syncytial virus activity and pneumococcal disease in infants: a time series analysis of US hospitalization data. PLoS Med. (2015) 12:e1001776. 10.1371/journal.pmed.1001776 25562317 PMC4285401

[5] de Steenhuijsen Piters WA Heinonen S Hasrat R Bunsow E Smith B Suarez-Arrabal MC. Nasopharyngeal microbiota, host transcriptome and disease severity in children with respiratory syncytial virus infection. Am J Respir Crit Care Med. (2016) 194:1104–15. 10.1164/rccm.201602-0220OC 27135599 PMC5114450

[6] Stark JM Stark MA Colasurdo GN LeVine AM. Decreased bacterial clearance from the lungs of mice following primary respiratory syncytial virus infection. J Med Virol. (2006) 78:829–38. 10.1002/jmv.20631 16628585

[7] Lundgren A Bhuiyan TR Novak D Kaim J Reske A Lu YJ. Characterization of Th17 responses to *Streptococcus pneumoniae* in humans: comparisons between adults and children in a developed and a developing country. Vaccine. (2012) 30:3897–907. 10.1016/j.vaccine.2012.03.082 22504663 PMC3369217

[8] Ampofo K Bender J Sheng X Korgenski K Daly J Pavia AT. Seasonal invasive pneumococcal disease in children: role of preceding respiratory viral infection. Pediatrics. (2008) 122:229–37. 10.1542/peds.2007-3192 18676537

[9] Lang PO Aspinall R. Can we translate vitamin D immunomodulating effect on innate and adaptive immunity to vaccine response? Nutrients. (2015) 7:2044–60. 10.3390/nu7032044 25803545 PMC4377899

[10] Sundaram ME Coleman LA. Vitamin D and influenza. Adv Nutr. (2012) 3:517–25. 10.3945/an.112.002162 22797987 PMC3649720

[11] Chen S Sims GP Chen XX Gu YY Chen S Lipsky PE. Modulatory effects of 1,25-dihydroxyvitamin D3 on human B cell differentiation. J Immunol. (2007) 179:1634–47. 10.4049/jimmunol.179.3.1634 17641030

[12] Chun RF Liu PT Modlin RL Adams JS Hewison M. Impact of vitamin D on immune function: lessons learned from genome-wide analysis. Front Physiol. (2014) 5:151. 10.3389/fphys.2014.00151 24795646 PMC4000998

[13] Camargo CA Jr Ganmaa D Frazier AL Kirchberg FF Stuart JJ. Randomized trial of vitamin D supplementation and risk of acute respiratory infection in Mongolia. Pediatrics. (2012) 130:e561–7. 10.1542/peds.2011-3029 22908115

[14] Laaksi I Ruohola JP Mattila V Auvinen A Ylikomi T Pihlajamaki H. Vitamin D supplementation for the prevention of acute respiratory tract infection: a randomized, double-blinded trial among young Finnish men. J Infect Dis. (2010) 202:809–14. 10.1086/654881 20632889

[15] Nowson CA McGrath JJ Ebeling PR Haikerwal A Daly RM Sanders KM. Vitamin D and health in adults in Australia and New Zealand: a position statement. Med J Aust. (2012) 196:686–7. 10.5694/mja11.10301 22708765

[16] Wei R Christakos S. Mechanisms Underlying the regulation of innate and adaptive immunity by vitamin D. Nutrients. (2015) 7:8251–60. 10.3390/nu7105392 26404359 PMC4632412

[17] Prietl B Treiber G Pieber TR Amrein K. Vitamin D and immune function. Nutrients. (2013) 5:2502–21. 10.3390/nu5072502 23857223 PMC3738984

[18] Olliver M Spelmink L Hiew J Meyer-Hoffert U Henriques-Normark B Bergman P. Immunomodulatory effects of vitamin D on innate and adaptive immune responses to *Streptococcus pneumoniae*. J Infect Dis. (2013) 208:1474–81. 10.1093/infdis/jit355 23922371

[19] Vissers M Habets MN Ahout IM Jans J de Jonge MI Diavatopoulos DA An in vitro model to study immune responses of human peripheral blood mononuclear cells to human respiratory syncytial virus infection. J Vis Exp. (2013) 82:e50766 10.3791/50766 PMC4047926 24379004

[20] Do LAH Tse R Nathanielsz J Anderson J Ong DS Chappell K. An improved and high throughput respiratory syncytial virus (RSV) micro-neutralization assay. J Vis Exp. (2019) 10.3791/59025 30741261

[21] Fitch N Becker AB HayGlass KT. Vitamin D [1,25(OH)2D3] Differentially regulates human innate cytokine responses to bacterial versus viral pattern recognition receptor stimuli. J Immunol. (2016) 196:2965–72. 10.4049/jimmunol.1500460 26895836

[22] Moffitt KL Gierahn TM Lu YJ Gouveia P Alderson M Flechtner JB. T(H)17-based vaccine design for prevention of *Streptococcus pneumoniae* colonization. Cell Host Microbe. (2011) 9:158–65. 10.1016/j.chom.2011.01.007 21320698 PMC3061323

[23] Moffitt KL Malley R Lu YJ. Identification of protective pneumococcal T(H)17 antigens from the soluble fraction of a killed whole cell vaccine. PLoS ONE. (2012) 7:e43445. 10.1371/journal.pone.0043445 22905267 PMC3419164

[24] Hoe E Nathanielsz J Toh ZQ Spry L Marimla R Balloch A. Anti-inflammatory effects of vitamin D on human immune cells in the context of bacterial infection. Nutrients. (2016) 8:806. 10.3390/nu8120806 27973447 PMC5188461

[25] Liang SC Tan XY Luxenberg DP Karim R Dunussi-Joannopoulos K Collins M. Interleukin (IL)-22 and IL-17 are coexpressed by Th17 cells and cooperatively enhance expression of antimicrobial peptides. J Exp Med. (2006) 203:2271–9. 10.1084/jem.20061308 16982811 PMC2118116

[26] Aujla SJ Chan YR Zheng M Fei M Askew DJ Pociask DA. IL-22 mediates mucosal host defense against Gram-negative bacterial pneumonia. Nat Med. (2008) 14:275–81. 10.1038/nm1710 18264110 PMC2901867

[27] Hoe E Boelsen LK Toh ZQ Sun GW Koo GC Balloch A. Reduced IL-17A secretion is associated with high levels of pneumococcal nasopharyngeal carriage in fijian children. PLoS ONE. (2015) 10:e0129199. 10.1371/journal.pone.0129199 26069966 PMC4466549

[28] Zhao F Hoechst B Gamrekelashvili J Ormandy LA Voigtlander T Wedemeyer H. Human CCR4+ CCR6+ Th17 cells suppress autologous CD8+ T cell responses. J Immunol. (2012) 188:6055–62. 10.4049/jimmunol.1102918 22615204 PMC3370143

[29] Brucklacher-Waldert V Steinbach K Lioznov M Kolster M Holscher C Tolosa E. Phenotypical characterization of human Th17 cells unambiguously identified by surface IL-17A expression. J Immunol. (2009) 183:5494–501. 10.4049/jimmunol.0901000 19843935

[30] Bluestone JA Abbas AK. Natural versus adaptive regulatory T cells. Nat Rev Immunol. (2003) 3:253–7. 10.1038/nri1032 12658273

[31] Sehrawat S Rouse BT. Interplay of regulatory T cell and Th17 cells during infectious diseases in humans and animals. Front Immunol. (2017) 8:341. 10.3389/fimmu.2017.00341 28421070 PMC5377923

[32] Antunes G Evans SA Lordan JL Frew AJ. Systemic cytokine levels in community-acquired pneumonia and their association with disease severity. Eur Respir J. (2002) 20:990–5. 10.1183/09031936.02.00295102 12412694

[33] Sommer A Fabri M. Vitamin D regulates cytokine patterns secreted by dendritic cells to promote differentiation of IL-22-producing T cells. PLoS ONE. (2015) 10:e0130395. 10.1371/journal.pone.0130395 26107738 PMC4480856

[34] Eklund D Persson HL Larsson M Welin A Idh J Paues J. Vitamin D enhances IL-1beta secretion and restricts growth of *Mycobacterium tuberculosis* in macrophages from TB patients. Int J Mycobacteriol. (2013) 2:18–25. 10.1016/j.ijmyco.2012.11.001 26785783

[35] Liu PT Schenk M Walker VP Dempsey PW Kanchanapoomi M Wheelwright M. Convergence of IL-1beta and VDR activation pathways in human TLR2/1-induced antimicrobial responses. PLoS ONE. (2009) 4:e5810. 10.1371/journal.pone.0005810 19503839 PMC2686169

[36] Marriott HM Gascoyne KA Gowda R Geary I Nicklin MJ Iannelli F. Interleukin-1beta regulates CXCL8 release and influences disease outcome in response to *Streptococcus pneumoniae*, defining intercellular cooperation between pulmonary epithelial cells and macrophages. Infect Immun. (2012) 80:1140–9. 10.1128/IAI.05697-11 22158745 PMC3294658

[37] Lin KL Sweeney S Kang BD Ramsburg E Gunn MD. CCR2-antagonist prophylaxis reduces pulmonary immune pathology and markedly improves survival during influenza infection. J Immunol. (2011) 186:508–15. 10.4049/jimmunol.1001002 21098218 PMC3723340

[38] Nguyen DT Louwen R Elberse K van Amerongen G Yuksel S Luijendijk A. *Streptococcus pneumoniae* enhances human respiratory syncytial virus infection in vitro and in vivo. PLoS ONE. (2015) 10:e0127098. 10.1371/journal.pone.0127098 25970287 PMC4430531

[39] Culley FJ Pennycook AM Tregoning JS Dodd JS Walzl G Wells TN. Role of CCL5 (RANTES) in viral lung disease. J Virol. (2006) 80:8151–7. 10.1128/JVI.00496-06 16873271 PMC1563837

[40] Klein M Obermaier B Angele B Pfister HW Wagner H Koedel U. Innate immunity to pneumococcal infection of the central nervous system depends on toll-like receptor (TLR) 2 and TLR4. J Infect Dis. (2008) 198:1028–36. 10.1086/591626 18700834

[41] Goritzka M Makris S Kausar F Durant LR Pereira C Kumagai Y. Alveolar macrophage-derived type I interferons orchestrate innate immunity to RSV through recruitment of antiviral monocytes. J Exp Med. (2015) 212:699–714. 10.1084/jem.20140825 25897172 PMC4419339

[42] Sadeghi K Wessner B Laggner U Ploder M Tamandl D Friedl J. Vitamin D3 down-regulates monocyte TLR expression and triggers hyporesponsiveness to pathogen-associated molecular patterns. Eur J Immunol. (2006) 36:361–70. 10.1002/eji.200425995 16402404

[43] Yuan FF Marks K Wong M Watson S de Leon E McIntyre PB. Clinical relevance of TLR2, TLR4, CD14 and FcγRIIA gene polymorphisms in *Streptococcus pneumoniae* infection. Immunol Cell Biol. (2008) 86:268–70. 10.1038/sj.icb.7100155 18180796

[44] Verma R Jung JH Kim JY. 1,25-Dihydroxyvitamin D3 up-regulates TLR10 while down-regulating TLR2, 4, and 5 in human monocyte THP-1. J Steroid Biochem Mol Biol. (2014) 141:1–6. 10.1016/j.jsbmb.2013.12.012 24373795

[45] Kim TH Lee HK. Innate immune recognition of respiratory syncytial virus infection. BMB Rep. (2014) 47:184–91. 10.5483/BMBRep.2014.47.4.050 24568879 PMC4163887

[46] Klein Klouwenberg P Tan L Werkman W van Bleek GM Coenjaerts F. The role of Toll-like receptors in regulating the immune response against respiratory syncytial virus. Crit Rev Immunol. (2009) 29:531–50. 10.1615/CritRevImmunol.v29.i6.40 20121698

[47] Zhao Y Yu B Mao X He J Huang Z Zheng P. Dietary vitamin D supplementation attenuates immune responses of pigs challenged with rotavirus potentially through the retinoic acid-inducible gene I signalling pathway. Br J Nutr. (2014) 112:381–9. 10.1017/S000711451400097X 24833277

[48] Kanno A Tanimura N Ishizaki M Ohko K Motoi Y Onji M. Targeting cell surface TLR7 for therapeutic intervention in autoimmune diseases. Nat Commun. (2015) 6:6119. 10.1038/ncomms7119 25648980

[49] Alvarez-Rodriguez L Lopez-Hoyos M Garcia-Unzueta M Amado JA Cacho PM Martinez-Taboada VM. Age and low levels of circulating vitamin D are associated with impaired innate immune function. J Leukoc Biol. (2012) 91:829–38. 10.1189/jlb.1011523 22345707

[50] Sande CJ Njunge JM Airway response to respiratory syncytial virus has incidental antibacterial effects. Nat Commun. (2019) 10:2218 10.1038/s41467-019-10222-z 31101811 PMC6525170

[51] Gupta A Sjoukes A Richards D Banya W Hawrylowicz C Bush A. Relationship between serum vitamin D, disease severity, and airway remodeling in children with asthma. Am J Respir Crit Care Med. (2011) 184:1342–9. 10.1164/rccm.201107-1239OC 21908411 PMC3471128

[52] Pletz MW Terkamp C Schumacher U Rohde G Schutte H Welte T. Vitamin D deficiency in community-acquired pneumonia: low levels of 1,25(OH)2 D are associated with disease severity. Respir Res. (2014) 15:53. 10.1186/1465-9921-15-53 24766747 PMC4046524

[53] Thorburn K Harigopal S Reddy V Taylor N van Saene HK. High incidence of pulmonary bacterial co-infection in children with severe respiratory syncytial virus (RSV) bronchiolitis. Thorax. (2006) 61:611–5. 10.1136/thx.2005.048397 16537670 PMC2104657

[54] Beadling C Slifka MK. How do viral infections predispose patients to bacterial infections? Curr Opin Infect Dis. (2004) 17:185–91. 10.1097/00001432-200406000-00003 15166819

[55] Madhi SA Klugman KP. A role for *Streptococcus pneumoniae* in virus-associated pneumonia. Nat Med. (2004) 10:811–3. 10.1038/nm1077 15247911 PMC7095883

[56] Dickie LJ Church LD Coulthard LR Mathews RJ Emery P McDermott MF. Vitamin D3 down-regulates intracellular Toll-like receptor 9 expression and Toll-like receptor 9-induced IL-6 production in human monocytes. Rheumatology. (2010) 49:1466–71. 10.1093/rheumatology/keq124 20435648

[57] Shen E Wang M Xie H Zou R Lin Q Lai L. Existence of Th22 in children and evaluation of IL-22 + CD4 + T, Th17, and other T cell effector subsets from healthy children compared to adults. BMC Immunol. (2016) 17:20. 10.1186/s12865-016-0158-8 27338754 PMC4918114

[58] Onwuneme C Molloy EJ. Question 2: vitamin D intake for preterm infants: how much do they really need? Arch Dis Child. (2018) 103:808–11. 10.1136/archdischild-2018-315363 29950354

