# Supplementary material for: Vitamin D Induces Differential Effects on Inflammatory Responses During Bacterial and/or Viral Stimulation of Human Peripheral Blood Mononuclear Cells
Source: Front Immunol. 2020 Apr 7;11:602. doi: 10.3389/fimmu.2020.00602 (PMC7154168; doi:10.3389/fimmu.2020.00602)
Supplement: Supplementary file 1 [file Data_Sheet_1.docx]

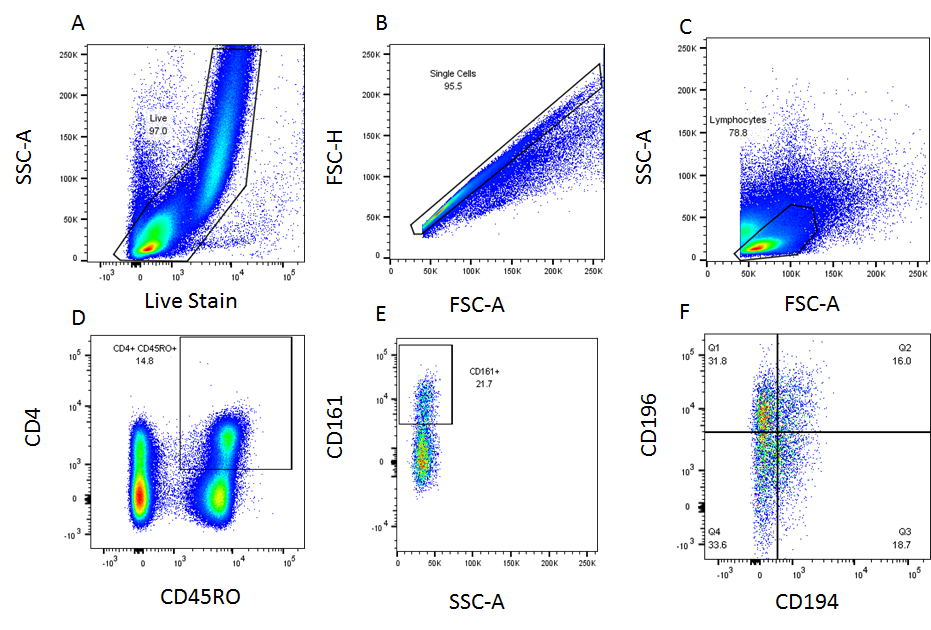


**Supplementary Figure 1:** The following gating strategy was used to obtain Th17 cells. Following live-dead gating (A) and singlets population gating (B), lymphocytes were determined by forward (FSC-A) and side (SSC-A) scatter (C) Based on lymphocytes, cells that were positive for CD4 and CD45RO were gated for (D) followed by CD161 positive gating (E). Lastly, CD161+ cells were further gated for CD194 and CD196 (F) indicating Th17 cells.

**
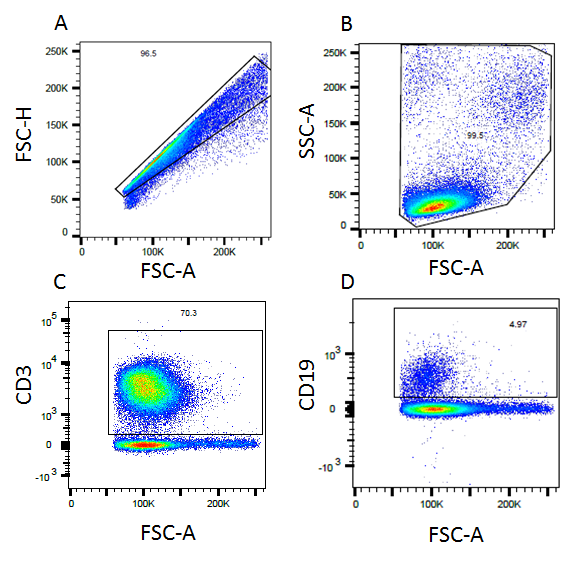
Supplementary Figure 2:** The following gating strategy was used to obtain T and B-cells. Based on a singlets population (A), lymphocytes were determined by FSC-A and SSC-A (B)**.** From lymphocytes, Cells that were CD3+ against FSC-A were T-cells (C) and cells that were CD19+ against FSC-A were B-cells (D).

**
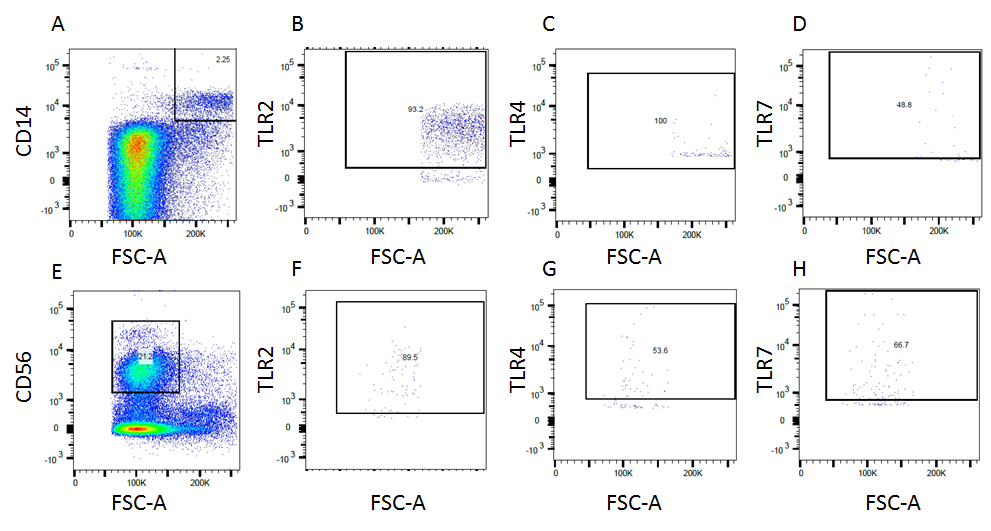
**

**Supplementary Figure 3:** The following gating strategy was used to obtain monocytes, NK cells and TLR2+, TLR4+ and TLR7+ expression. From lymphocytes (described in Supplementary Figure 2), cells that were CD14+ against FSC-A were monocytes (A). Then TLR2+, TLR4+ and TLR7+ were plotted against FSC-A to obtain their expression on CD14+ cells (B-D). Cells that were CD56+ against FSC-A were considered NK-cells (E) and TLR expression was gated for in a similar manner to those in CD14+ cells (F-H).


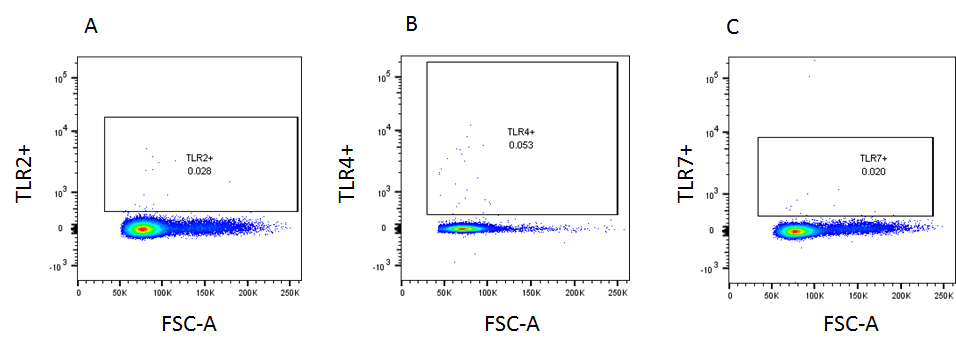


**Supplementary Figure 4:** Representative fluorescence minus one (FMO) staining for TLR expression on lymphocytes (A-C) as this was the lowest frequency cell population studied. The gating strategy was similar to those shown in Supplementary Figures 1, 3.
